# CORAL—Catamaran for Underwater Exploration: Development of a Multipurpose Unmanned Surface Vessel for Environmental Studies

**DOI:** 10.3390/s24144544

**Published:** 2024-07-13

**Authors:** Luca Cocchi, Filippo Muccini, Marina Locritani, Leonardo Spinelli, Michele Cocco

**Affiliations:** 1 Istituto Nazionale di Geofisica e Vulcanologia, Via di Vigna Murata 605, 00143 Rome, Italy; filippo.muccini@ingv.it (F.M.); marina.locritani@ingv.it (M.L.); 2 Edgelab S.r.l, Via Privata OTO, 10, 19136 La Spezia, Italy; leonardo.spinelli@edgelab.eu (L.S.); michele.cocco@edgelab.eu (M.C.)

**Keywords:** unmanned surface vehicle, side scan sonar, seabed morphology, acoustic imagery, biological habitat, coastal environment

## Abstract

CORAL (Catamaran fOr UndeRwAter expLoration) is a compact, unmanned catamaran-type vehicle designed and developed to assist the scientific community in exploring marine areas such as inshore regions that are not easily accessible by traditional vessels. This vehicle can operate in different modalities: completely autonomous, semi-autonomous, or remotely assisted by the operator, thus accommodating various investigative scenarios. CORAL is characterized by compact dimensions, a very low draft and a total electric propulsion system. The vehicle is equipped with a single echo-sounder, a 450 kHz Side Scan Sonar, an Inertial Navigation System assisted by a GPS receiver and a pair of high-definition cameras for recording both above and below the water surface. Here, we present results from two investigations: the first conducted in the tourist harbour in Pozzuoli Gulf and the second in the Riomaggiore-Manarola marine area within the Cinque Terre territory (Italy). Both surveys yielded promising results regarding the potentiality of CORAL to collect fine-scale submarine elements such as anthropic objects, sedimentary features, and seagrass meadow spots. These capabilities characterize the CORAL system as a highly efficient investigation tool for depicting shallow bedforms, reconstructing coastal dynamics and erosion processes and monitoring the evolution of biological habitats.

## 1. Introduction

The exploration of the seafloor and water column stands as a significant and pivotal scientific endeavour aimed at comprehending the evolution of our oceans and their complex interplay with the global rise in temperature [1] and the anomalous fluctuations in water mass circulation [2]. These phenomena are intricately linked to the overarching issue of ongoing climate change [3,4]. Investigating the present dynamics of the ocean and scrutinizing each of its components in detail (seabed, water column or interactions with the atmosphere) is imperative for gaining insights into the future trajectory of our planet over the next 10–20 years. 

The analysis and description of seabed morphology provide crucial insights into the geological evolution of hidden portions of the Earth, including the continental shelf, deep basins, and intriguing volcanic seamounts associated with submarine magmatic manifestations. The exploration of ocean morphology traces its origins back to the early 1960s, marked by the development of pioneering single-beam acoustic sonars. 

Today, swath bathymetry is commonly acquired using multibeam systems capable of examining large areas simultaneously by emitting multiple beams synchronously from a single acoustic transmitter. Moreover, the Side Scan Sonar (hereafter SSS) system proves invaluable in the classification of bedforms (acoustic classification) and identifying anthropic objects. This instrumentation records the backscatter from the acoustic waves emitted by two lateral sonars, providing detailed insights into both the morphology and the seabed response to the acoustic sounding. Backscatter represents the intensity value (dB) of the acoustic wave reflected by the seafloor and recorded by the sonar. This parameter provides the reconstruction of seafloor typology, distinguishing hard bottoms or anthropic objects (resulting in high backscatter) from soft bottoms, such as sand, mud and ripple formations (associated with low scatter) [5]. The advantage of SSS lies in its dual spatial coverage, with two transducers mounted on the port and starboard sides of the towfish, enabling surveys in close proximity to the seabed at varying depths. Commonly, marine morphologic surveys are conducted using ships or research vessels. However, over the last three decades, oceanographic operations have been increasingly assisted by unconventional payloads such as Unmanned Surface Vessel (USV) or Autonomous Underwater Vessel (AUV) [6,7,8,9]. These vessels are typically capable of operating autonomously, with minimal or no direct involvement of surveyors. Similar to aerial applications, USVs can be likened to marine drones. Their compact size and high-performance capabilities in terms of geographical positioning enable them to conduct morphological investigations in very shallow waters or close to the coast, reaching areas that may not be easily accessible to conventional research vessels. 

In recent years, the unmanned marine vehicle market has witnessed significant growth, marked by the development of highly sophisticated systems capable of conducting extended operations, including geophysical investigations, direct inspections, and water sampling for periods exceeding 24 h. These unmanned systems cover substantial regional portions of the sea, spanning hundreds of nautical miles. Moreover, the simultaneous operation of multiple USVs enhances coverage and efficiency, ultimately reducing survey times [10].

A notable example of a high-performance USV system is Saildrones, a 7 m long unmanned sailing boat equipped with large solar panels that provide the power supply for prolonged investigations lasting over 12 months [11]. Another noteworthy unmanned vehicle is Fugro Blue Essence, a surface vehicle that competes favourably with conventional human-manned research vessels in terms of acquiring bathymetric data [12].

In this study, we describe the capabilities of CORAL (Catamaran fOr undeRwAter expLoration), a sea surface prototype catamaran designed to investigate coastal marine areas in both assisted mode (with an operator) and complete autonomous mode. The proposed system belongs to a family of small USVs (greater than 2–3 m in total length) designed for scientific study. The primary goal of CORAL is to provide an autonomous, open-access platform with a customizable navigation system for any type of scientific investigation of the seabed and water column. CORAL does not aim to compete with other commercial USVs that have higher performance in terms of speed and resolution but they do not not allow for complete customization of the navigation and control systems.

CORAL, compared to similar-class autonomous vehicles, has dimensions that allow it to host various types of sensors for both morphological and chemical/biological studies. Additionally, the reduced draft, the zero-emission electric propulsion and the minimal acoustic noise make it extremely versatile for all scientific investigation operations in shallow marine areas. One of its main objectives is the study of the coastal marine portion, which has depths between 0 and 5 m and is notoriously difficult to investigate with traditional research vessels.

The CORAL vehicle is equipped with a single echo-sounder, an Inertial Navigation System (INS) assisted by a GPS receiver, a pair of high-definition cameras for recording both above and below the water surface, and a 450 kHz SSS system mounted on a pole. Furthermore, this system can be improved with additional specific payloads such as a CTD probe (conductibility, temperature and depth), magnetic sensors and/or marine litter detection sensors to enhance its versatility and functionality. 

The system offers the capability to visualize and pre-process backscatter images directly from the ground station, enabling real-time validation of the data. CORAL is designed to conduct interdisciplinary surveys in crucial coastal areas, as exemplified by real-case studies of the Pozzuoli harbour and the survey of the Riomaggiore-Manarola inshore sector within the Cinque Terre marine protected area, Italy. This latter site exhibits a distinctive seabed morphology characterized by abrupt shoals and rocky outcrops that make it a challenge to investigate effectively by using conventional craft. CORAL’s adaptability and advanced features make it well suited for addressing the complexities of such coastal environments.

## 2. Materials and Methods

### 2.1. The Vessel Design

CORAL is a compact, unmanned catamaran-type vehicle designed and developed to assist the scientific community in exploring marine areas such as inshore regions (harbours, small lakes and ponds), which are not easily accessible by traditional ships or crafts. This USV consists of two primary fiberglass hulls with a low draft, accompanied by two main electric thrusters for both propulsion and manoeuvring. Detailed dimensions and technical specifications can be found in Table 1.

**Figure 1:**
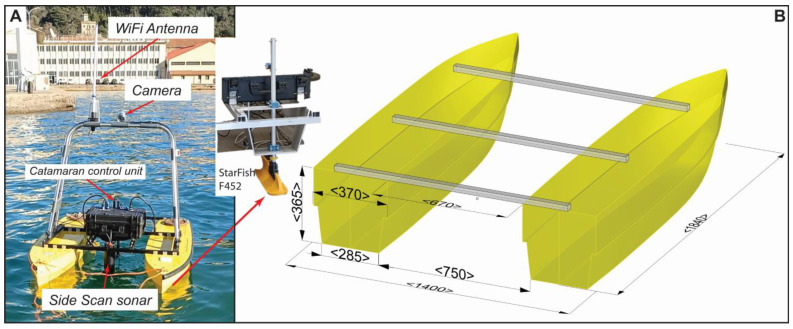
(**A**) Image of the CORAL system during a sea trial; the image of the Starfish F452 SSS installed on a sliding pole is reported as an inset. (**B**) CAD representation of the catamaran hulls (prospecting view); quotes are in millimetres.

The primary structure of the vessel is composed of two hulls connected by three main steel poles, forming a catamaran-like configuration with an overall length of 184 cm and a total breadth of 140 cm (Figure 1). Each hull is assembled with a 4mm thin laminate (lay-up) using two distinct fiberglass layers, namely, MAT 300 and BIAX 600, denoting glass fabrics of 300 g/m^2^ and biaxial 600 g/m^2^, respectively. The layering sequence involves MAT 300, BIAX 600, BIAX 600 and MAT 300. The hull design originated from the NPL (National Physical Laboratory) series [13], typical for high-speed displacement vessels, and was subsequently customized to meet the displacement vs. total length ratio. After calculating the hydrodynamic water resistance values (total effective) outlined in Table 2, we studied a propulsion system capable of maintaining an average cruise speed of approximately 3 knots with a maximum speed of 5 knots. The primary propulsion is achieved through the utilization of two brushless motors with total throttling capabilities for both forward and reverse movements. These motors are paired with propellers featuring a diameter of 100 mm, each capable of generating 200 W of power (3.72 kgf). The propulsion is supplied by two 12 V, 100 Ah LiPo4 battery packs placed inside the two hulls. The thruster control signals are pulse-width modulated and come from the Main Navigation Unit electronic board (hereafter MNU), which is galvanically isolated from the battery packs. This is obtained by means of a couple of factory-made opto-isolator breakout boards specifically designed to prevent high-current feedback. Any failure or damage that may occur to the motors in the hull section does not affect the MNU.

The three steel poles connecting the two hulls also function as a support structure for the waterproof case housing the main electronics and communication tools. Additionally, a roll bar fixed to the steel poles accommodates the Wi-Fi antenna, GPS module, and IP camera (Figure 1A). Antennas, sensors and other auxiliary components are connected to the navigation motherboard through dedicated cabling and waterproof connectors. This design ensures that each component of the USV can be easily disassembled and replaced, if necessary, facilitating maintenance and adaptability.

### 2.2. System Overview

**Scheme 1:**
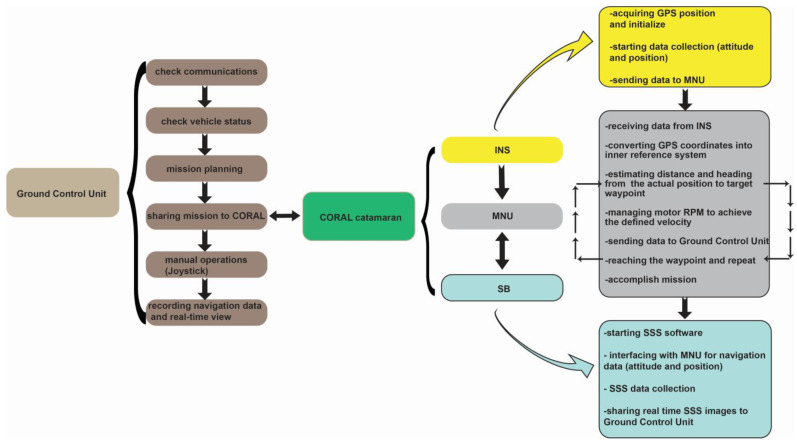
Integrated workflow of the CORAL system during an operative mission.

CORAL is not merely a vehicle; rather, it is conceptualized as a system comprising four key subsystems as follows: (i) the main vessel with an on-board MNU (deputed to command and control the vessel), (ii) a ground control unit featuring a tailored Graphical User Interface (hereafter GUI), (iii) the sensor board (hereafter SB) connected to scientific instrumentation, and (iv) the communication unit. This architecture necessitates a specific workflow that interactively engages all these components (see Scheme 1).

Primarily, the navigation is overseen by the ground control unit, through which users can design the mission, specifying waypoints and run lines. This information is then shared through a peer-to-peer Wi-Fi link with the on-board navigation computer. This computer device manages the thrusters to navigate along the predefined run lines (reaching in sequence the designated waypoints). Simultaneously, the on-board unit relays real-time vessel position information back to the ground station for a continuous evaluation of the status of the navigation.

During the mission, users have the flexibility to activate the SSS and the underwater camera, enabling real-time observation of the data through the ground control unit. The subsequent sections provide a detailed description of each individual component within the CORAL system.

#### 2.2.1. Command and Control Subsystem

The command and control subsystem is placed inside a waterproof case above the CORAL catamarans. This major component includes the following devices:

- MNU board.
- SB.
- INS coupled to a GPS receiver.
- Ethernet switch.
- Thruster controllers.
- Opto Isolator boards and battery measurement system.
- 24 V LiFePO4 16 Ah.

**Figure 2:**
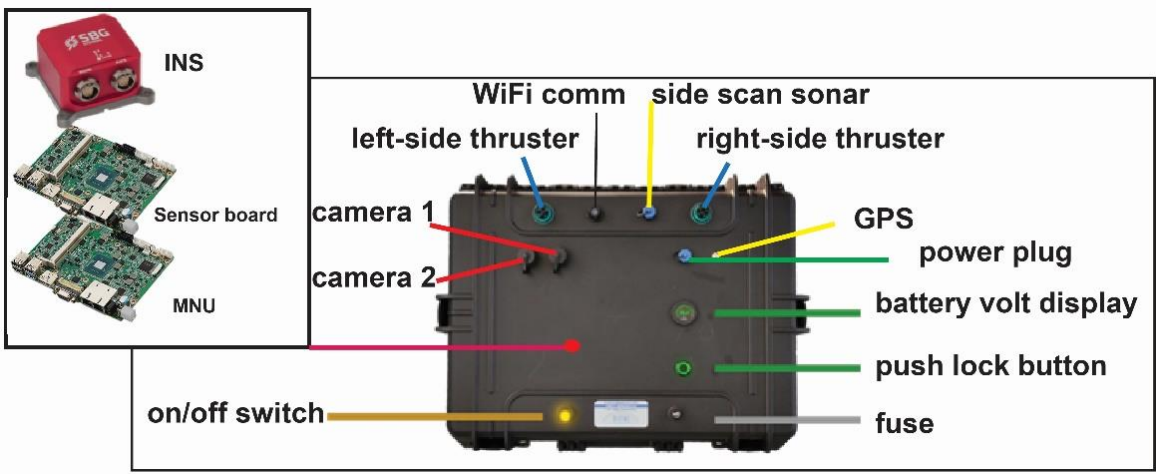
Image of the waterproof case installed on board the catamaran and hosting the electronics, mainboards and INS system.

The waterproof case has been conceptualized as a standalone unit; it can be detached from the catamaran and managed separately in cases of mobilization and/or substitution of several components. On the front panel, the ON/OFF button and related fuse holder can be found, along with a round display showing the battery status and voltage that is activated only when triggered by the respective push lock button. Further above, the waterproof external connectors are laid out: a pair of connectors dedicated to the signals for port and starboard thrusters, as well as the connectors for the echo sounder and the SSS. In addition, there are connectors designed for the external multi-constellation GPS antenna, IP Camera and lastly, the battery charger (Figure 2).

- Upon power-up, the system enters “initializing status”, during which the unit activates a short-range 2.4 GHz Wi-Fi access point, enabling direct access to the MNU and SB for data download and/or upload of custom configuration. After approximately 5 min, if there is no active connection from other devices, the short-range link is disabled as part of the battery management system. The waterproof case contains the primary electronics, including an Ethernet switch, the INS, and the two motherboards, MNU and SB. The electronics and motherboards are powered by a secondary battery pack (24 V 16 Ah), separated from the main power source used for propulsion. This setup allows the maximum energy supply to be dedicated to the individual systems, thereby avoiding mutual interference.

The MNU is a Linux-based embedded computer with a built-in 256 GB SSD and 8 GB of RAM (MIO5251 motherboard, Advantech Ltd., Taiwan). It is equipped with two Ethernet interfaces and four serial ports, supporting both RS232 and 422/485 protocols. This unit serves as the host for the primary navigation software (refer to the following section) and oversees the data received from the INS via a RS232 port. Its primary function is to control the main propulsion system and fulfils the sea surface navigation requirements communicated by the ground control unit. 

The SB is a stand-alone Windows-based board dedicated to managing the SSS system. This board acquires raw sonar data through a USB port and interfaces with the INS (and GPS) via a serial port. The SB sends real-time sonar data to the ground station and simultaneously receives the configuration instances by using the Wi-Fi communication channel. Specifications for both boards are shown in Table 3.

The SBG Ellipse mod-E INS represents the core of the CORAL navigation system. This high-performance sensor interprets, collects and shares with the MNU the data related to the dynamics of the vessels: pitch roll, heave, heading, velocity and acceleration along three main directions. The SBG Ellipse is composed of three industrial-grade high-performance accelerometers coupled to three MEMS (Micro-Electro Mechanical Systems) gyroscopes and a three-axial anisotropic magneto-resistive magnetometer. The device is able to collect data at a maximum rate of 200 samples per second (Hz). Notably, the INS also serves as a unique timing source essential for the internal synchronization of various sensors. By establishing a direct connection with a GPS receiver, the INS rectifies the geographical positioning of the vessel. As widely acknowledged, vessel movements such as roll and pitch can introduce discrepancies in positioning between the GPS receiver and the vessel’s centre of mass (where the INS is typically located). This discrepancy, often of metric magnitude, is primarily proportional to the separation distance between the two points and is compounded by the inherent uncertainty of the GPS. The INS effectively mitigates this bias, ensuring the accurate geographical positioning of the vessel. Additionally, thanks to the inner Kalman filter algorithm, the inertial sensor permits precise navigation even in the absence of GPS data (i.e., dead reckoning navigation).

#### 2.2.2. Scientific Sensors Payload

While CORAL has been engineered to assist in the acquisition of diverse scientific data, its foremost objective is to furnish information about the seafloor. This includes detailed morphological features, identifying anthropic objects, and mapping specific biological patterns, such as meadows of *Posidonia Oceanica* or *Cymodocea nodosa*. The direct exploration of the seabed is conducted using an IP underwater camera (Barluscam model), strategically positioned at the centre of the vessel, approximately midway between the two hulls. The camera is submerged about 20 cm below sea level and is configured with a downward orientation. However, users can manually adjust its inclination angle to achieve various observation perspectives. Equipped with 3 white lights plus 4 infrared lights, each emitting 1000 lumens with ten levels of adjustable brightness, the camera facilitates near-field observations. Despite this, identifying the nature of the seafloor using the camera, especially in the far-field case, poses a challenge due to the limited penetration of the light in the water. Optimal resolution is mostly achieved in clear water environments.

Beside the direct camera observation, CORAL explores the seafloor by integrating a single beam echo sounder and a SSS. Broadly speaking, a single-beam sensor stands out as one of the most prevalent and cost-effective tools utilized in bathymetric surveys, especially in the case of USV applications. It enables the representation of the seabed morphology of a survey area by collecting a series of individual soundings. This sensor functions by emitting a singular acoustic wave and subsequently recording the reflected wave from the seabed. By analysing the travel time and knowing the sound velocity in the water medium, the single-beam sensor can accurately compute the distance between the sensor and the seabed, providing essential bathymetric information. In our case, we use a 115 kHz single-beam echo sounder featuring a 30° beam width and a measurement range of 30 m. The sensor is installed on the pole of the underwater camera and connected to the MNU. The bathymetric measurements are collected by the navigation software and merged with the INS-GPS information. Considering the limited range, this sensor is suitable for investigation of very shallow marine areas (ports and/or docks).

The vehicle is also equipped with the Starfish F452 sonar system by BluePrint Subsea, situated on the aft side. The SSS works at a frequency of 452 kHz and features Chirp technology. This technique involves specific frequency modulation of the emitted acoustic wave, effectively increasing the signal-to-noise ratio and thus enhancing the resolution of the resulting sonar images. The Starfish F452 has a horizontal beam width equal to 0.8° and a maximum lateral coverage of 100 m. The sonar is installed on a sliding pole placed between the two hulls (Figure 1). This setup enables the adjustment of the immersion of the sensor in a range of 20–40 cm below sea level.

The SSS is directly linked to the dedicated motherboard (SB) via a USB cable, on which the specialized acquisition software (Starfish Scanline V2.1.2; https://www.blueprintsubsea.com/starfish/support accessed on 10 May 2024) is installed and actively controlled by the operator. Essential data, such as GPS coordinates and attitude information (pitch, roll, heading and velocity), crucial for the optimal acquisition of SSS data, are communicated from the MNU to the SB through RS232 interfaces (Scheme 1). This seamless integration ensures the synchronized operation of the various components, facilitating accurate and comprehensive data acquisition during underwater explorations. A live view of the SSS streamline and real-time adjustments to acoustic parameters, including gain, contrast, and lateral swath coverage, can be executed by remotely accessing the SB through the ground control unit. This feature allows operators to dynamically optimize the sonar imaging parameters during exploration, enhancing flexibility and adaptability in response to varying underwater conditions. The acquired raw data are stored inside the SB and shared with other devices using Wi-Fi communication or by accessing the board directly.

### 2.3. Software Implementation

The CORAL navigation software was developed in a ROS (Robotic Operating System) framework [14,15], an open-access and multiplatform system that enables the implementation of routines, low-level device control, and I/O data sharing. ROS (we used ROS Melodic Morenia distribution) supports various programming languages, including C++, Python 2 and Lisp (ROSLisp). The ROS code is primarily organized into packages, which can be viewed as program routines containing one or more nodes and configuration files. Nodes are processes capable of computation and communication with other nodes. The primary workflow between different nodes is centred on the subscription-publication concept of a specific topic. A node that subscribes to a topic requests connections from another node that publishes that topic, thus establishing a connection over an agreed-upon connection protocol. The most commonly used protocol in ROS is TCPROS, which follows the standard TCP/IP sockets.

**Scheme 2:**
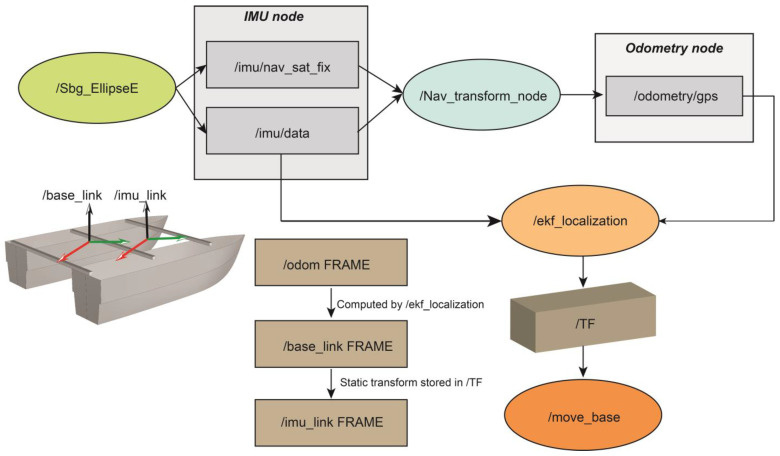
ROS workflow of the multi-process CORAL navigation software.

The main software package custom-made for the CORAL project is called the “catamaran package” (Scheme 2). It comprises source files designed to interpret velocity commands for the thrusters in a differential drive configuration, acquire voltage readings from motors and MNU battery packs, configure parameters, and provide launch files tailored for various situations.

In accordance with the ROS standard, velocity messages are expressed in the *geometry_msgs/Twist* format, consisting of two vectors containing linear (x, y and z) and angular velocities. These messages are read by the PCA node (library for servo controlling) within the catamaran package, which subscribes to the *cmd_vel* topic. Subsequently, the messages are converted into values for the left and right thrusters and transmitted via the I2C interface to a PCA8695, which generates PWM (pulse width modulation) signals for the Electronic Speed Controllers (ESCs). Velocity commands can be published to the *cmd_vel* topic either by users in remote navigation mode or by the *move_base* node during autonomous navigation. In remote navigation mode, inputs from joysticks or gamepads are converted into *geometry_msgs/Twist* messages by the *teleop_twist_joy* package, which then publishes them to the *cmd_vel* topic (an example of the code for this node is reported in Supplementary Materials).

Sensor data are managed by specific nodes within a multi-process architecture that grants better stability against potential failures. A key node of the architecture is the sbg ellipseE, which is part of the /*sbg_driver* package available on both the ROS Wiki and the official SBG Systems GitHub profile (https://github.com/SBG-Systems, accessed on 10 February 2021). The *sbg_ellipseE* node provides the acquisition of navigation data from INS and their publication onto specific topics that carry custom formats. Upon configuration, the INS can be set so it publishes also ROS standard messages into dedicated topics such as */imu/data* for quaternion, angular and linear velocities and */imu/nav_sat_fix* for latitude, longitude and altitude, making it easier to interface with other packages. These data are expressed in *sensor_msgs/Imu* message format, containing three vectors: quaternions, linear accelerations and angular velocities (see example of message reported in Supplementary Materials).

One of the crucial tasks of navigation software is to manage the position of the vehicle by executing a transformation from the robot’s world frame to the real-world position. CORAL has two main reference frames inside the vehicle, as follows: /*base_link* and /*imu_link*. The first represents the frame rigidly attached to the centre of the vehicle, while the second is a frame geometrically linked to the position of the INS within the vehicle, and it cannot be exactly aligned with the /*base_link* frame (see Scheme 2). The two reference systems differ by a simple static geometric offset. This offset is stored and broadcasted by a specific node named */tf*. The same node takes care of another reference frame named /*odom,* which is used to track the position of the vehicle in the geographical real world.

The */tf* node broadcasts and manages the geometrical relations between each frame, but the transformation between /*odom* and /*base_link* is performed using an extended Kalman filter algorithm that resides in the */ekf_localization* node.

As the GPS acquires the first fix, /*odom* is generated at the same location as the /*base_link*. Additionally, the /*navsat_transform_node* starts converting GPS data (latitude and longitude) into positions in the /*odom* frame (x and y), and using INS data provides an estimated odometry message that includes position and velocities in the robot’s world frame. Position, velocities and heading data are fused by the /*ekf_localization* node, which updates the relation between /*odom* and /*base_link*.

Autonomous navigation is achieved through the specific package *move_base*. It utilizes the transformation tree to coordinate self-governing guidance, following a waypoint-based navigation approach. Initially, the routine transforms the latitude and longitude coordinates of predefined waypoints into the robot’s world reference system, loading them sequentially. A dedicated process calculates the angles and distances between the vehicle position and the waypoint, adjusting the heading direction accordingly to the position of the waypoint to reach. The *move_base* node also provides a control loop for navigation status at a high frequency (20 Hz), sending back information about velocity, distance and heading. The target is reached if the position of the vessel is within a tolerance circular area of about 3 m in diameter centred on the selected waypoint (see pseudocode in Supplementary Material). This tolerance parameter can be defined by the user, considering several factors: sea state, level of survey accuracy, and the main purpose of the survey (for instance, in the case of SSS investigation, the large swath of the sonar can allow a larger tolerance). Once the waypoint is reached, the system proceeds to the next one. On the contrary, if the assigned waypoint is not achieved, the system attempts a set of recovery manoeuvres, like moving backward, rotating in place to find the correct heading and trying to reach the target position again. After five unsuccessful attempts, the system aborts the mission.

### 2.4. Communication System and Ground Control Unit

The exchange of information between the catamaran and the ground station, and vice versa, is ensured through a direct 2.4/5 GHz Wi-Fi communication channel. This wireless link is performed using a couple of Ubiquity Dual-Band airMAX^®^ AC Radio Bullet transmitters configured in “point-to-point” link mode. This device implements a proprietary protocol named airMAX that allows each interlocutor to send and receive data using pre-designated time slots scheduled by an intelligent AP controller. This methodology effectively eliminates hidden node collisions and optimizes airtime efficiency, leading to a significant reduction in latency and improved noise immunity. The receivers provide an output power of approximately 21 dBm, with a maximum data throughput of over 300 Mbps (at 5 GHz).

CORAL incorporates two paired 9 dBi omnidirectional antennas: one installed on the vessel and the other on the ground station unit. This configuration guarantees real-time camera views and facilitates information exchange between the mobile and fixed units within a maximum range of 2 km. We carried out navigation tests during rainy weather conditions without finding any decrease in performance. However, the distance range provided by the Wi-Fi antenna can significantly decrease as a function of the multiple effects of various sea state conditions, such as rough seas and high sea waves, which can cause radio wave reflectivity on the sea surface with sensible loss of information. In addition, possible adverse weather conditions, such as rainfall or storms, can reduce the capability of Wi-Fi communication. For these reasons, the CORAL system will be further enhanced with a 4G router to facilitate data sharing in case of Wi-Fi channel failure or to extend the distance range beyond 2 km.

**Figure 3:**
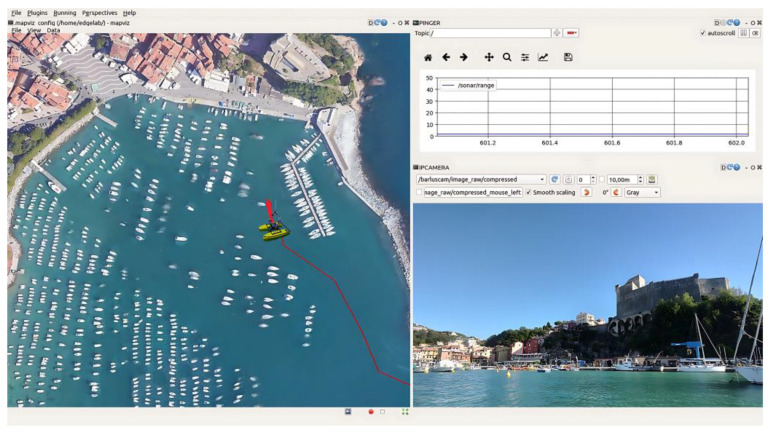
Snapshot of the CORAL GUI installed on the ground control station. The left tile displays tracks of USV navigation overlaid on a geographical background. In the upper right tile, the depth of seafloor acquired by the echo sounder is shown. The lower right tile provides a real-time streaming view from the navigation camera.

Considering various applications and operative scenarios, the type of ground control station can range from a standard notebook to a rugged laptop or smartphone. The Wi-Fi link provides a network where multiple devices can be connected simultaneously. In specific situations, it is preferable to operate with two separate laptops: one dedicated to navigation where the GUI is installed, and another device managing scientific data acquisition. The GUI is derived from a suite of tools within the ROS system named RQT. This framework enables the integration of different plugins and dockable windows into a single interface. The CORAL’s GUI has been specifically customized and built to show the navigation information and primary commands to control the vehicle. Although it is possible to redesign its appearance in real time, the default configuration, stored in the ground station, activates the frontal IP Camera streaming (underwater optional), the real-time readings of the echo-sounder altimeter, and the GPS tracking on a map (Figure 3). A set of tiled maps can be previously downloaded and stored at the ground station, ensuring the availability of a geographical background during the acquisition even without active internet coverage.

The connection between the control station and the vehicle is established through a distributed computing environment. The catamaran serves as the ROS master, enabling multiple machines to interact with it as long as they are connected to the same network and properly identified. Once connected, these machines can share nodes and topics, which will inherit the name of the advertiser. Behind the double-click icon of the ground station GUI lies a complete ROS network setup system, facilitating bidirectional communication between the user and the vehicle.

The GUI typically operates on the Ubuntu 18.04 distribution to ensure compatibility with ROS Melodic, which must match the version installed on the vehicle. However, specific bridge tools are available to facilitate communication between different ROS versions. Additionally, CORAL can be controlled using Android tablets and smartphones via an app called ROS-Mobile. In this case, users can manoeuvre the vehicle using a simulated joystick and access the camera view, similar to the functionality available in the RQT GUI.

## 3. Results

In this section, we present the findings from two distinct SSS investigations conducted utilizing the CORAL system. The first survey focused on mapping the seabed morphology of a commercial and touristic harbour located in Pozzuoli Bay (Naples, Italy). We include this example to showcase the CORAL survey capability of collecting precise data within confined areas characterized by shallow waters (minimum depth of 2 m) and the presence of boats and obstacles.

The second focused on a segment of the coastal marine area situated in front of Riomaggiore, a village within the Cinque Terre La Spezia (Italy), located in the easternmost part of the Ligurian Gulf. In this instance, our data underscore the capability of the CORAL system to distinguish various fine-scale depositional features, thereby facilitating the classification of the seabed along the shoreline into distinct macroareas.

In both cases, the collected raw sonar datasets were processed using Hips and Sips 11.1 software by Caris Teledyne (https://www.teledynecaris.com/en/home; accessed on 10 August 2023) The processing of data was performed according to the following workflow:

(1) Converting binary property files recorded by the Starfish Scanline acquisition software into XTF (Extended Triton Format) files, which are readable by Hips and Sips software.
(2) Georeferencing the various track lines composing the survey and correcting the navigation by removing intervening spikes and portions of the line affected by high acoustic noise (e.g., turning points).
(3) Applying the sonar imagery correction creating a beam pattern. This is unique and computed for each survey, and it introduces an across-track bias that needs to be removed from the imagery.
(4) Checking the imagery along the tracks and adjusting the gain and colour palette. During this step, high-noise portions of soundings can be removed. Additionally, selected data can be corrected by applying despeckle filtering. This algorithm estimates the acoustic intensity inside a 3 × 3 pixels moving windows, replacing that value with the average of the surrounding pixels.
(5) Creating the mosaic for the entire survey. The individual sonar images acquired along the track lines are then merged together using a weighted average blending filter.

### 3.1. Pozzuoli Harbour

In June 2023, we conducted a test survey in Pozzuoli Bay, focused on a small section of a commercial/touristic docking area. Given the presence of boats and obstructions, we operated the CORAL system in assisted mode. In this configuration, the system follows specific fixed heading directions until the operator initiates a new directional command instance via ground station.

Within the study area, the water depth ranges from 4 m in the central canal to 10/12 m near the entrance of the port. Consequently, the swath of the SSS changed from 45 m to 25 m as we passed from the outer to the inner side of the port, respectively.

**Figure 4:**
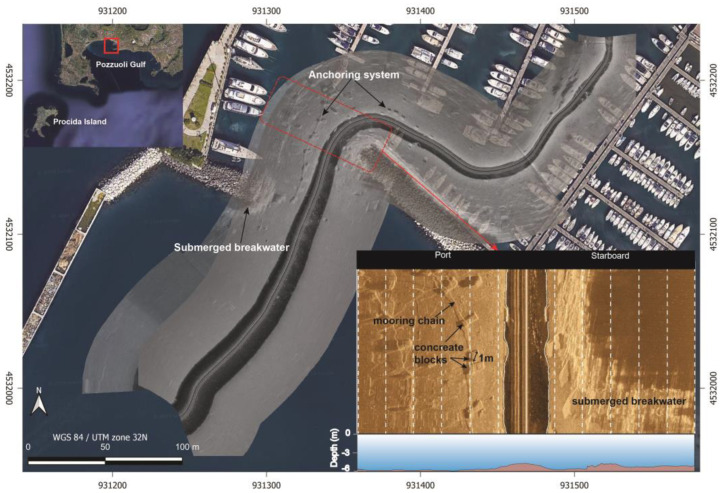
SSS mosaic of Pozzuoli harbour (location in the upper-left inset). Details of the acoustic signature of the submerged anchoring system (red rectangle) are reported as inset in the right-side panel.

In this scenario, the SSS acquisition was facilitated in real-time by the operator, who adjusted parameters such as swath distance and sounding gain during data sampling. We conducted two separate sonar-sounding passages: initially, moving from South to North, and then, in the opposite direction, traversing two breakwaters that laterally delimited the entrance of the port. Subsequently, we collected data near the floating docks, where a large number of boats are moored. The purpose of this survey was to identify submerged anchoring systems in the docking area and assess the CORAL system’s ability to detect small anthropic objects placed on the seabed. In Figure 4, we present the sonar mosaic with a resolution of 10 cm/pixel, overlaid on the satellite image of the study area. The sonar image distinctly identifies the presence of a breakwater system composed of groups of boulders of metric dimensions. These constructions exhibit a specific sonar signature characterized by high acoustic reflectivity, displaying a scattered ray pattern generated by the accumulation of hard materials (solid rock portions) with multiple reflective surfaces. Additionally, sonar data accurately depicted the submerged anchoring system where floating docks and boats are moored. In the inset of Figure 4, we observe detailed seabed sonar soundings over the laid-mooring area. Here, we observe the presence of aligned cubic-shape concrete blocks (occasionally joined together) measuring approximately 1 m per side, connected by a long mooring chain. The resolution of the investigation allows us to easily detect objects with sub-metric dimensions. Furthermore, the lateral investigation conducted by the SSS enables the acquisition of information beneath the floating docks and in between the boat moorings, areas inaccessible to other direct measurements.

### 3.2. Riomaggiore Site

Riomaggiore and Manarola are two villages located within the Cinque Terre, situated within the La Spezia province. This region is renowned for its picturesque fishing villages perched atop steep cliffs, each connected to small harbours by a labyrinth of narrow alleys.

Over recent decades, the Cinque Terre region has witnessed an exponential increase in tourism, leading to a consistent demand for additional infrastructure and accommodations, resulting in increased land utilization along the coastline [16,17,18]. The coastal area of Riomaggiore is characterized by a high susceptibility to hydrogeological instability. In the past two decades, occurrences of instability such as collapses and landslides have markedly escalated in both frequency and intensity [19], significantly altering the coastline morphology and causing its gradual retreat towards the hinterland [20]. 

Geologically, the Riomaggiore area shows a bedrock primary formed by sandstone and claystone flysch dated to the Upper Oliogocene (Macigno Formation, Tuscan Unit), which also outcrops in the inner land. Moving northward, in between Manarola and Corniglia towns, the stratigraphic sequence changes with the outcropping of the Canetolo Formation (sub-liguride unit), which is mostly made up of claystones intercalated with carbonates, with ages ranging from the Late Cretaceous to the Eocene [21,22,23]. This area experienced intense compressional tectonics starting in the Miocene, resulting in a multiphase deformation of the sedimentary sequences with the emplacement of a main antiform fold (named La Spezia fold) with an axis trending in the NW-SE (N 150°) direction and a set of NE-SW strike slip faults [24].

The survey conducted in the coastal marine area between Riomaggiore and Manarola in 2022 using the CORAL system provided new insights into the seafloor morphology and sedimentary features of the region (Figure 4). This area is the southwestern portion of the larger Cinque Terre Marine Protected area that encompasses the coastal strip between Riomaggiore and Monterosso village (i.e., https://www.parconazionale5terre.it/Earea-marina-protetta.php accessed on 10 May 2024). In this area, the coastal biodiversity and the extraordinary variety of animal and plant species are strongly safe-guarded, limiting and forbidding any anthropic activities (seabed mining, marine transportation, and fishing). In this context, CORAL can represent a vehicle suitable for research in this kind of protected marine area. 

From a sedimentary point of view, the survey area is characterized by a mixture of fine sediments alternating with coarse marine deposits, indicative of inshore sedimentary processes. Additionally, the presence of large blocks and boulders near the shoreline suggests the occurrence of partial flank collapses or minor slides from the steep coastal cliffs.

The objectives of the survey were twofold. First, it aimed to evaluate the performance of the CORAL system in open sea conditions, assessing its ability to navigate in the presence of currents, wind, and varying sea states. Second, the survey provided a snapshot of the seabed morphology to understand the evolution of the depositional and sedimentary systems over time. 

**Figure 5:**
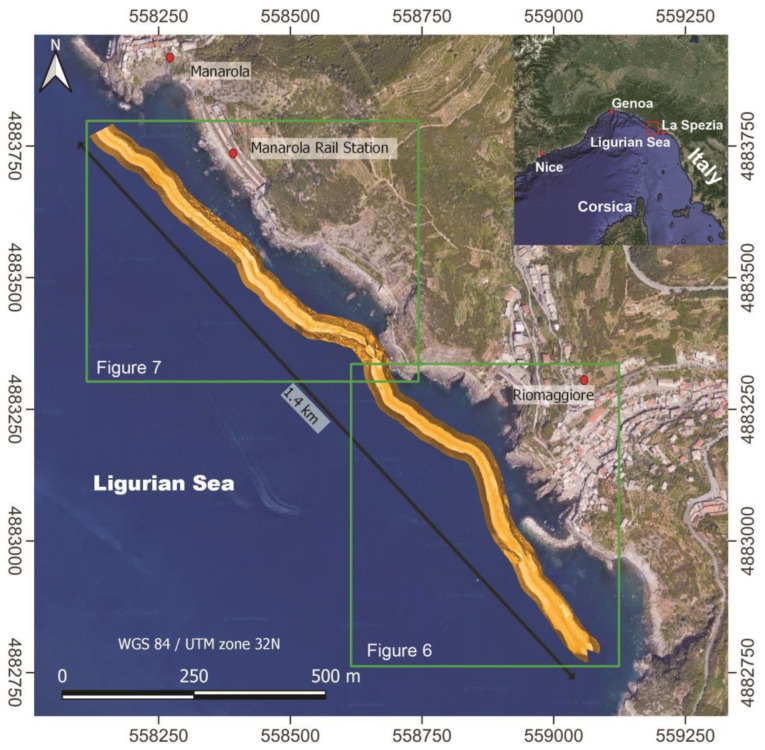
SSS survey of the Riomaggiore-Manarola marine area. The large area has been divided into two subfigures (green rectangles) for the further interpretation of seabed morphology (see Figure 6 and Figure 7).

The SSS survey was conducted following NW-SE track lines, largely parallel to the shorelines. In the first 50 m of the coastal strip, the seafloor shows a slight-to-moderate morphologic gradient with variation of the water depth in a range of 6–13 m, with peaks of 1.5/2 m and, in some instances, the emergence of large rocks. These rapid changes in water depth posed significant challenges for data collection. To address these challenges, a fixed lateral swath of 25 m was utilized for the sonar soundings. However, some zones were not surveyed due to the presence of obstacles such as boats or other obstructions. Additionally, for safety reasons, areas with depths shallower than 1.5 m were not covered by the survey. The results of the sonar investigation are reported in Figure 5 (a mosaic with 15 cm/pixel resolution).

**Figure 6:**
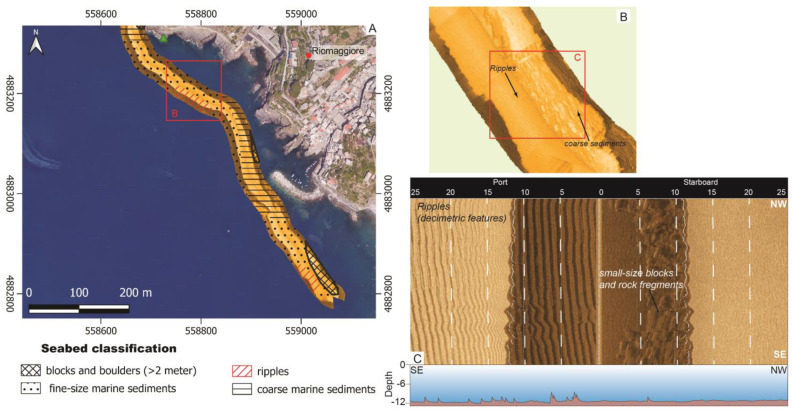
(**A**) Overview of SSS investigation of the SE domain of the Riomaggiore survey with overlaid seabottom characterization obtained from acoustic data analysis. (**B**) Snapshot of a sonar mosaic related to a specific area showing the ripple bedforms interrupted by coarse deposits. (**C**) Same area as (**B**) directly observed on the Starfish Scanline V2.1.2 software interface.

**Figure 7:**
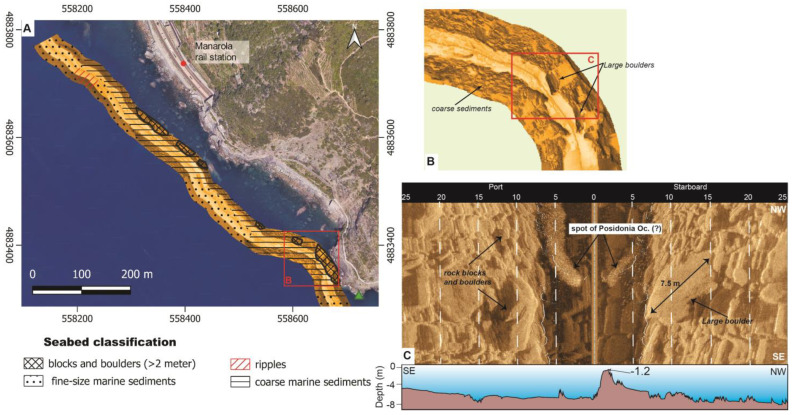
(**A**) Overview of SSS investigation of the NW domain of the Riomaggiore-Manarola survey with overlaid seabottom characterization obtained from acoustic data analysis. (**B**) Snapshot of a sonar mosaic related to a specific area showing blocks and boulders. (**C**) Same area as (**B**) directly observed on the SSS software interface; here specific acoustic signatures related to spots of biological habitat are observed as well.

We conducted a classification of the major seabed morphological and sedimentary features within the surveyed area, dividing it into two distinct sub-regions: a southeastern area limited to Riomaggiore harbour and beach (Figure 6) and a second northwestern region encompassing the Manarola marine area (Figure 7). In both areas, we identified four main sedimentary and depositional features: (1) fine marine sediments, (2) ripple features, (3) coarse marine products characterized by decimetre-to-meter-sized clasts and rock fragments, and (4) rock blocks and boulders larger than (at least) 2 m. In Figure 6B,C, we present a detailed SSS image of a seabed marked by ripple formations. These exhibit straight-to-sinuous bedforms with decimetre heights, indicating a preferential or unidirectional flow of the main currents affecting the area. Such bedforms are sporadic and interrupted by coarse deposits.

As we move northward, the seafloor features undergo a transition characterized by an increase in coarse sediments and the presence of large rock fragments and boulders. These elements are concentrated, particularly between the Manarola rail station and the promontory. This area has witnessed numerous instability events involving the collapse of massive blocks, rocks, and debris. Due to the high risk of instability, the pedestrian/hiking trail connecting Riomaggiore and Manarola municipalities, known as “Via dell’Amore”, has been closed since 2012 [25].

The SSS image (Figure 7) provides detailed insights into the distribution of these rockfall-like deposits, featuring large boulders exceeding 7/8 m in dimension (Figure 7B,C). Among these large rock fragments, we observe peculiar features characterized by a dotted-like acoustic signature (Figure 7C), which can represent spots of biological habitat like seaweed *s.l* or *Posidonia Oceanica*, each spanning a few meters in size. Large *P. Oceanica* meadows are present further northward, particularly near Monterosso village [26] and in other locations within the Cinque Terre marine protected area.

## 4. Discussion and Conclusions

The continuous advancement in marine technologies has enabled the exploration of new investigative methodologies, increasingly geared towards remote and autonomous approaches where human involvement is primarily focused on supervising and interpreting data acquisition. The advantages of this approach are manifold: it allows for the investigation of otherwise inaccessible areas, enhances spatial resolution with minimal human intervention, facilitates the planning of long-lasting campaigns with precise navigation, and reduces the likelihood of errors in repeatability. Furthermore, it mitigates the risks associated with various types of investigations in hazardous environments, such as those affected by volcanic or tectonic activity or prone to intense instability such as rockfalls.

In this context, we present and discuss the capabilities of CORAL, a small autonomous catamaran designed for investigating shallow marine areas. We stressed the system by conducting a series of survey tests at various sites with different characteristics of seabed morphology. In our initial investigation, we tested the capability of CORAL to navigate in shallow and highly trafficked marine areas, such as tourist harbours. In this scenario, the system demonstrated its ability to collect SSS data with remarkable precision, successfully detecting small objects even in inaccessible areas, such as those located below floating docks.

In the second test, we conducted an acoustic sonar study of the seabed morphology in the Riomaggiore-Manarola marine area within Cinque Terre, Italy. The sonar data yielded valuable insights into the distribution of seabed sediments with high precision and efficiency. We were able to detect fine-scale sedimentary features such as ripples as well as spots characterized by biological habitats. The detailed depiction of the bedforms and the classification of the lithologies are crucial information for understanding coastal dynamics, erosion processes, and habitat mapping. Furthermore, by comparing data from this survey with historical data, we can track changes in the seafloor morphology, providing insights into the long-term evolution of the coastal environment in the Cinque Terre marine protected area.

The CORAL system has specific features that make it extremely interesting and versatile for marine investigation of coastal and port marine areas. This type of vehicle, based on an open platform, can be considered a prototype and belongs to the class of scientific-oriented USV systems [6,9,27,28]. Its main strengths lie in its navigation system, which is based on the ROS platform and open-access libraries, as well as its high modularity. The engineering of the system incorporates various modules (i.e., subsystems) that can be integrated with each other or, alternatively, excluded as needed. For instance, the scientific payload can be easily removed or replaced with other payloads without requiring any modifications to the main navigation system.

Another strength of CORAL is its high-displacement fiberglass hull. The engineering design of the hull’s shape and the choice of material allow for smooth navigation in open waters as well. Generally, small marine USVs featuring a monohull or catamaran shape are made of ABS or polyethylene (i.e., [9,27]), materials that significantly reduce the weight of the hull but do not allow for navigation in rough waters. The limitation of small marine USVs is their poor capability to handle open waters, restricting their use to limited water areas (lakes and/or bays). The CORAL fiberglass hull enables the system to be employed in several operational scenarios compared to other similar USV systems. 

On the other hand, we can critically discuss the current limitations of CORAL. The system is still in the prototype stage, although it is in an advanced phase. The vehicle offers versatile navigation, but the software currently does not integrate high-level SLAM algorithms that could enhance its performance. Additionally, an AI-based mapping of seabed features could be included to improve the results of traditional acoustic investigations [27]. Considering these criticisms, we are continuously adopting new approaches and technologies to improve both navigation and survey capabilities. The major planned changes for the near future include: (1) incorporating an obstacle avoidance system based on camera view and LiDAR to enhance the safety of navigation and implement a related SLAM algorithm; (2) installing solar panels to increase its endurance, thereby extending its operational capabilities; (3) coupling a 4G router with the Wi-Fi antenna to improve the robustness of data communication; and (4) integrating additional scientific sensors such as a high-frequency multibeam and/or a marine litter detecting sensor, hence orienting its applicative field also to the water column studies.

## Figures and Tables

**Table 1:** Dimensions and technical specifications of the CORAL vehicle.

**Length overall (LOA)**	1840 mm
**Width of a single hull**	370 mm
**breadth overall (BOA)**	1400 mm
**Draft**	180 mm
**Weight of the hulls**	60 kg
**Total displacement**	115 kg
**Propulsion**	2 × brushless thrusters, 12 V (205 W)
**Maximum speed**	5 knots
**Autonomy**	8 h (average speed: 3 knots)
**Power supply (propulsion)**	2 × 12 V 180 Ah 2160 Wh
**Addition of power supply (main electronics and sensors)**	1× lithium, 24 V, 16 Amp/h
**Communication**	Point-to-point 2.4/5 GHz Wi-Fi and/or 4G LTE
**Camera**	2× IP Camera (Barlus, Shenzen, China )
**Side scan sonar**	Starfish 452F (Blueprint Subsea, Ulverston, UK )–450 KHz nominal frequency
**Single echo-sounder**	Blue Robotics Ping Sonar, measurement range: 50 m
**Navigation system**	INS–SBG Ellipse-E with external GPS antenna

**Table 2:** Estimated values of total resistance (Rt, in Newton) and effective power (Pe, in Watt) of CORAL catamaran.

Velocity (Knt)	Rt (N)	Pe (W)
0.5	0.727	0.221
1	1.949	1.004
1.5	8.496	6.973
2	12.827	13.196
2.5	19.512	25.946
3	27,521	42,472
3.5	37,208	68,142
4	79,931	164,477
4.5	126,062	295,373
5	147.166	378.536
5.5	156.979	448.695
6	162.408	501.295
6.5	162.327	547.453
7	166.597	599.926

**Table 3:** Technical specifications of the two main motherboards managing the navigation software and scientific sensors.

Specification	Main Navigation Unit (MNU)	Sensor Board (SB)
Model	MIO 5251J-U0A1E (Advantech Ltd., Taiwan)	RCO-1010
CPU	Intel Celeron J1900 2 GHz (Quad-Core)	Intel Celeron J1900 4C
RAM	8 GB DDR3L, 1333 MHz	8 GB DDR3 1600 MHz
I/O interfaces	RS-232/485/422, USB3.0, I2C, SMBus, GPIO, Ethernet	RS-232/484/422, USB3.0, Ethernet
Power	Single 12 V DC power input	9–48 VDC
Power consumption	0.62 A @12 V (7.44 W)	0.62 A @12 V (7.44 W)
Dimensions (L × W × H)	146 × 102 × 19 mm	150 × 49 × 105
Weight	0.5 kg	0.8 kg

## Data Availability

Data are available from the corresponding author on reasonable request.

## Supplementary Materials

The following supporting information can be downloaded at: https://www.mdpi.com/article/10.3390/s24144544/s1.
