# Supplementary material for: CORAL—Catamaran for Underwater Exploration: Development of a Multipurpose Unmanned Surface Vessel for Environmental Studies
Source: Sensors (Basel). 2024 Jul 13;24(14):4544. doi: 10.3390/s24144544 (PMC11280589; doi:10.3390/s24144544)
Supplement: Supplementary file 1 [file sensors-24-04544-s001.zip › sensors-3028478-supplementary.pdf]

Supplementary materials

CORAL - Catamaran fOr UndeRwAter expLoration: development of a multipurpose Unmanned Surface Vessel for environmental studies

Luca Cocchi [1,*], Filippo Muccini[1], Marina Locritani[1], Leonardo Spinelli [2] and Michele Cocco [2]

[1] Istituto Nazionale di Geofisica e Vulcanologia, Via di vigna murata 605, Rome, Italy
[2] Edgelab S.r.l, Via privata OTO, 10, La Spezia, Italy
* Correspondence: luca.cocchi@ingv.it

Supplementary Text 1

Below is an example of code used to manage the main thrusters. The script creates a ROS node named "motor_controller" and interfaces with I2C boards. The node acts as a subscriber to the topic */cmd_vel* with a message type of *geometry_msgs/Twist*.

```
int main(int argc, char **argv)

{

// Create a node named motor_controller declaring a custom signal handler for emergency stop

ros::init(argc, argv, "motor_controller", ros::init_options::NoSigintHandler);

// Initialize management of the node

ros::NodeHandle n;

// Declare a 10Hz rate of update

ros::Rate rate(10);

// The variable status will be used to determine the success or fail of the read and write operations through I2C hardware interface

uint32_t status;

// Initialize the SUSI library required to access the I2C hardware, wrong execution of this function is reflected on the status variable

status = SusiLibInitialize();

// Execute the function set_prescaler which sets the update frequency of the PWM board to 50Hz

set_prescaler();

// Execute the init function

init();

// Inform user that the node is ready

printf("Set up completed...\n");
```

```
signal(SIGINT, mySigintHandler);

//* The node act as a subscriber on the topic /cmd_vel with a message type geometry_msgs/Twist.h, the buffer is 5 element long, this to avoid unwanted behavior in case of erroneous stocking of messages.*/

ros::Subscriber sub = n.subscribe("cmd_vel", 5, motorCallback);

//While the node is active

while (n.ok())

{

//Check weather too much time has passed since the last received command

  if ((ros::WallTime::now().toSec() - last_received_message) > watchdog_timeout)    {

     // If stopped is false

     if (stopped == 0)

                {

                printf("EMERGENCY STOP!\n");

                // Execute the stop function

                stop();

                // commute stopped to true

                stopped = 1;

                }

  }

  ros::spinOnce();

}

// Continue to cycle

ros::spin();

return 0;

}
```

Supplementary Text 2

Here we provide the structure and main syntax of some ROS standard messages used in the navigation software. Refer to the main text for their specific applications.

1) **geometry_msgs/msg/Twist**

raw message definition:

# This expresses velocity in free space broken into its linear and angular parts.

```
Vector3  linear
float64 x
float64 y
float64 z

Vector3  angular
float64 x
float64 y
float64 z
```

2) **sensor_msgs/Imu**

raw message definition:

```
Header header

geometry_msgs/Quaternion orientation
float64 orientation_covariance # Row major about x, y, z axes

# This represents an orientation in free space in quaternion form.
float64 x
float64 y
float64 z
float64 w

geometry_msgs/Vector3 angular_velocity
float64 angular_velocity_covariance # Row major about x, y, z axes

# This represents a vector in free space.
float64 x
float64 y
float64 z

geometry_msgs/Vector3 linear_acceleration
float64[9] linear_acceleration_covariance # Row major x, y z

# This represents a vector in free space.

float64 x
float64 y
float64 z
```

3) sensor_msgs/NavSatFix

# Navigation Satellite fix for any Global Navigation Satellite System
# Specified using the WGS 84 reference ellipsoid

Header header

# satellite fix status information
NavSatStatus status

# Latitude [degrees]. Positive is north of equator; negative is south.
float64 latitude

# Longitude [degrees]. Positive is east of prime meridian; negative is west.
float64 longitude

# Altitude [m]. Positive is above the WGS 84 ellipsoid
# (quiet NaN if no altitude is available).
float64 altitude

# Position covariance [m^2] defined relative to a tangential plane
# through the reported position. The components are East, North, and
# Up (ENU), in row-major order.
# Beware: this coordinate system exhibits singularities at the poles.

float64 position_covariance

# If the covariance of the fix is known, fill it in completely. If the
# GPS receiver provides the variance of each measurement, put them
# along the diagonal. If only Dilution of Precision is available,
# estimate an approximate covariance from that.

uint8 COVARIANCE_TYPE_UNKNOWN = 0
uint8 COVARIANCE_TYPE_APPROXIMATED = 1
uint8 COVARIANCE_TYPE_DIAGONAL_KNOWN = 2
uint8 COVARIANCE_TYPE_KNOWN = 3
uint8 position_covariance_type

Supplementary Text 3

We report a pseudocode explaining the navigation software for CORAL catamaran. In particular, this example code relates to the *move_base* ROS node, used to estimate the distance and direction between the local position of the catamaran and the target waypoint. This portion of the code consists of two main functions: *move_to_waypoint* and *activate_recovery_behavior*.

The first function, *move_to_waypoint*, calls a subroutine to determine the exact geographic position and heading of the catamaran and computes the distance to the waypoint, updating data every 20 times per second (20 Hz). The routine is successfully accomplished if the distance between the vessel and the waypoint is less than 1.5 m, or in other words, if the vessel is within a circle of 3 m in diameter. If the attempt fails, the software initiates a second routine named *activate_recovery_behavior.* This latter involves specific manoeuvres to force the catamaran to reach the target position: first, the vessel moves backward for 5 m (current heading-180), then it rotates in place to determine the exact heading direction, and finally, it moves forward to reach the exact waypoint position. This function allows up to 5 attempts; after that, the mission is considered aborted.

```
INITIALIZATION
 Initialize ROS node "move_base "
 Load parameters (velocity, heading, tolerance, recovery_behavior)
 FUNCTION main()
  Set waypoint with x, y coordinates
  Set tolerance_area to 3 meters in diameter
  Set initial attempt to 0
  MAX_ATTEMPTS = 5
  WHILE attempt < MAX_ATTEMPTS
   result = move_to_waypoint(waypoint) //  estimation of  direction and distance to waypoint
   IF result == SUCCESS
    BREAK
   ELSE
    attempt = attempt + 1
    activate_recovery_behavior(waypoint) //acting  manoeuvres to reach the waypoint

  IF attempt == MAX_ATTEMPTS
  END IF
 END FUNCTION


 FUNCTION move_to_waypoint(waypoint)
  Start move_base action client
```

```
// Step 1: Check current heading of CORAL
current_heading = get_current_heading()
// Step 2: Estimate heading from local position to waypoint
current_position = get_current_position()
target_heading = calculate_heading(current_position, waypoint)
// Step 3: Estimate distance to waypoint
distance_to_waypoint = calculate_distance(current_position, waypoint)
// Adjust heading to face the waypoint
adjust_heading(target_heading)
// Send goal to move_base with waypoint coordinates
send_goal_to_move_base(waypoint)
// Frequency control loop at 20 Hz
rate = ROS.Rate(20)
result = FAILURE

WHILE not move_base goal achieved AND result != MOVE_BASE_SUCCESS
  Check current CORAL position
  distance_to_waypoint = calculate_distance(current_position, waypoint)
  IF distance_to_waypoint <= (tolerance_area / 2)
    result = MOVE_BASE_SUCCESS
    RETURN SUCCESS
  END IF
  Check move_base status
  IF move_base status == FAILURE
    result = MOVE_BASE_FAILURE
    RETURN FAILURE
  END IF
  // Sleep to maintain 20 Hz loop
  rate.sleep()
END WHILE
IF result != MOVE_BASE_SUCCESS
  RETURN FAILURE
```

```
  END IF
END FUNCTION

FUNCTION activate_recovery_behavior(waypoint)
  current_position = get_current_position()
  current_heading = get_current_heading()
  // Calculate the position 5 meters back along the current heading
  recovery_position = calculate_recovery_position(current_position, current_heading, 5)

  // Move to the recovery position
  move_to_position(recovery_position)
  current_heading = get_current_heading()
  target_heading = calculate_heading(current_position, waypoint)
  adjust_heading(target_heading)

  // Attempt to reach the waypoint again
  result = move_to_waypoint(waypoint)
  IF result == SUCCESS
    RETURN
  ELSE
    FOR angle FROM 0 TO 360 STEP 10
      Rotate to angle
      result = move_to_waypoint(waypoint)
      IF result == SUCCESS
        RETURN
      END IF
    END FOR
  END IF
END FUNCTION

FUNCTION wait_for_result()
  WHILE not move_base goal achieved
```

```
    Check current CORAL position
    distance_to_waypoint = calculate_distance (current_position, waypoint)

    IF distance_to_waypoint <= (tolerance_area / 2)
      RETURN MOVE_BASE_SUCCESS
    END IF

    Check move_base status
    IF move_base status == FAILURE
      RETURN MOVE_BASE_FAILURE
    END IF
  END WHILE
END FUNCTION

FUNCTION calculate_distance (position1, position2)
  dx = position2.x - position1.x
  dy = position2.y - position1.y
  distance = sqrt(dx*dx + dy*dy)
  RETURN distance
END FUNCTION

FUNCTION calculate_heading(position, waypoint)
  dx = waypoint.x - position.x
  dy = waypoint.y - position.y
  heading = atan2(dy, dx) // Calculate heading in radians
  RETURN heading
END FUNCTION

FUNCTION get_current_heading()
  // Retrieve the current heading of the CORAL from sensors or state
  RETURN current_heading_value
END FUNCTION
```

```
FUNCTION get_current_position()
  // Retrieve the current position of the CORAL from localization system
  RETURN current_position_value
END FUNCTION

FUNCTION adjust_heading(target_heading)
  // Rotate the CORAL to face the target heading
  WHILE abs(current_heading - target_heading) > tolerance
    Rotate towards target_heading
    current_heading = get_current_heading()
  END WHILE
END FUNCTION

FUNCTION send_goal_to_move_base(waypoint)
  // Send the waypoint as a goal to the move_base action client
  move_base_client.send_goal(waypoint)
END FUNCTION

FUNCTION calculate_recovery_position(current_position, current_heading, distance)

  // Calculate the new position 5 meters back along the current heading
  recovery_x = current_position.x - distance * cos(current_heading)
  recovery_y = current_position.y - distance * sin(current_heading)
  RETURN (recovery_x, recovery_y)
END FUNCTION

FUNCTION move_to_position(position)
  // Move the CORAL to the specified position
  move_base_client.send_goal(position)
  wait_for_result()
END FUNCTION
```

Call main()

END
